# Topical Application of Cinnamaldehyde Promotes Faster Healing of Skin Wounds Infected with *Pseudomonas aeruginosa*

**DOI:** 10.3390/molecules24081627

**Published:** 2019-04-25

**Authors:** Thiago A.F. Ferro, Eliene B. Souza, Mariela A.M. Suarez, João F.S. Rodrigues, Domingos M.S. Pereira, Saulo J.F. Mendes, Laoane F. Gonzaga, Márcia C.A.M. Machado, Maria R.Q. Bomfim, João B. Calixto, Jack L. Arbiser, Valério Monteiro-Neto, Eunice André, Elizabeth S. Fernandes

**Affiliations:** 1 Programa de Pós-Graduação, Universidade CEUMA, São Luís 65075-120, MA, Brazil; thafeitosaf@hotmail.com (T.A.F.F.); eliene.b-s@hotmail.com (E.B.S.); m-suarez86@hotmail.com (M.A.M.S.); joaofranciscosr@hotmail.com (J.F.S.R.); domingosmagno2@hotmail.com (D.M.S.P.); saulo.mendes@ceuma.br (S.J.F.M.); laoane_freitas@hotmail.com (L.F.G.); mcammachado@hotmail.com (M.C.A.M.M.); mrqbomfim@yahoo.com.br (M.R.Q.B.); valerio.monteiro@ceuma.br (V.M.-N.); 2 Centro de Inovação e Ensaios Pré-Clínicos-CIEnP, Florianópolis 88056-000, SC, Brazil; joao.calixto@cienp.org.br; 3 Department of Dermatology and Veterans Administration Medical Center, School of Medicine, Emory University, Atlanta, NY 30322, USA; jarbise@emory.edu; 4 Centro de Ciências da Saúde, Universidade Federal do Maranhão, São Luís 65080-805, MA, Brazil; 5 Departamento de Farmacologia, Universidade Federal do Paraná, Curitiba 81531-980, PR, Brazil; andreeu@hotmail.com

**Keywords:** cinnamaldehyde, *Pseudomonas aeruginosa*, skin wound, wound healing

## Abstract

Wound healing can be delayed following colonization and infection with the common bacterium *Pseudomonas aeruginosa*. While multiple therapies are used for their treatment, these are ineffective, expensive, and labour-intensive. Thus, there is an enormous unmet need for the treatment of infected wounds. Cinnamaldehyde, the major component of cinnamon oil, is well known for its antimicrobial properties. Herein, we investigated the effects of sub-inhibitory concentrations of cinnamaldehyde in the virulence of *P. aeruginosa*. We also assessed its healing potential in *P. aeruginosa*-infected mouse skin wounds and the mechanisms involved in this response. Sub-inhibitory concentrations of cinnamaldehyde reduced *P. aeruginosa* metabolic rate and its ability to form biofilm and to cause haemolysis. Daily topical application of cinnamaldehyde on *P. aeruginosa*-infected skin wounds reduced tissue bacterial load and promoted faster healing. Lower interleukin-17 (IL-17), vascular endothelial growth factor (VEGF) and nitric oxide levels were detected in cinnamaldehyde-treated wound samples. Blockage of transient receptor potential ankyrin 1, the pharmacological target of cinnamaldehyde, abrogated its healing activity and partially reversed the inhibitory actions of this compound on VEGF and IL-17 generation. We suggest that topical application of sub-inhibitory concentrations of cinnamaldehyde may represent an interesting approach to improve the healing of *P. aeruginosa*-infected skin wounds.

## 1. Introduction

A skin wound can be defined as a disruption or break of the skin barrier. Acute wounds are normally resolved in a timely manner, whilst chronic ones present with slow healing phases. Depending on the lesion extension, a wound can be classified as clean/simple (when there is minimal loss of tissue and healing occurs within 48 h after lesion) or complicated (when there is the loss of a large tissue area and a slow healing process is present). Healing [1] involves vascular (vasoconstriction followed by vasodilation) and inflammatory responses; the latter are characterized by plasma extravazation and leukocyte influx to the site of injury. Then, healing progresses into a proliferation phase in which connective tissue and novel vessels (granulation tissue) are formed; during this phase, the wound contracts and closes, forming a scar. Finally, during the maturation phase, the blood flow reduces and the scar is remodelled, becoming stronger by collagen deposition.

Skin wounds, especially those of a chronic or complicated nature, represent a major cause of morbidity and mortality, particularly in lower extremities. Wounds can become infected by bacteria, especially in patients in intensive care units and patients with different morbidities, including diabetes and poor skin perfusion [1,2]. Infection may result in a biofilm-containing non-growing bacteria encased in a mucoid coat that stimulates inflammation [1]. In this context, the inflammation-induced vascular leakage provides nutrients to the bacteria. Therefore, both the bacteria and the increased host vascular permeability contribute to delaying wound healing, resulting in a chronic wound phenotype [1]. Indeed, re-epithelialisation of the wound cannot occur until biofilm-induced inflammation is removed.

Although the management of wounds costs billions of dollars yearly, there is no universally effective method for their treatment [1,3]. Current clinical interventions include surgical debridement of lesions, complex dressings including alginate, foams, and silver, and hyperbaric oxygen [1,3]. Of importance, systemic antibiotics and topical antimicrobials may be administered when bacterial infection is present; however, they are of limited efficacy as, in this scenario, bacteria are not reproducing and the biofilm limits exposure to antimicrobials. 

*P. aeruginosa* is a biofilm-forming bacterium frequently detected in skin wounds, especially in deeper regions of the wound beds [4,5]. Of note, wounds infected by this microorganism are characterized by larger areas of lesion and a delayed healing process [6]. This, associated with the fact that *P. aeruginosa* presents both intrinsic and acquired antibiotic resistance [7,8], makes the clinical management of wounds infected by this pathogen a great challenge. Therefore, there is a great unmet need for inexpensive agents that can disrupt *P. aeruginosa* biofilm and, at the same time, promote wound healing. 

In this context, plant-derived compounds have potential as both antimicrobial and healing agents. Cinnamaldehyde, the major compound of the essential oil from *Cinnamomum* sp. stem barks, is well known for its ability to increase skin blood flow and for its antimicrobial properties against different bacteria including *P. aeruginosa*. These properties have been shown in different studies [9,10,11,12,13,14,15,16]; however, there are few reports of its healing effects [17,18]. 

A study by Takasao et al. [17] showed that the in vitro incubation of cinnamaldehyde with human skin fibroblasts induces collagen synthesis. More recently, this compound was found to stimulate human endothelial cell proliferation in vitro [18]. The same study demonstrated that the systemic administration of cinnamaldehyde in rats accelerates the healing of cutaneous wounds by inducing angiogenesis in the wounded area; however, the topical effects of this compound have not yet been addressed.

Herein, we investigated the in vitro antimicrobial actions of cinnamaldehyde against *P. aeruginosa* strains. The in vivo healing potential of the topical application of cinnamaldehyde on skin excision wounds infected or not with *P. aeruginosa*, as well as the mechanisms involved in this response, were also investigated in mice.

## 2. Results

### 2.1. Cinnamaldehyde Is Antimicrobial against P. aeruginosa Strains

The antimicrobial effects of cinnamaldehyde were initially assessed in ATCC standard and clinical isolates of *P. aeruginosa*. The strains showed different susceptibility profiles to clinically available antibiotics (Table 1). The isolate *P. aeruginosa* 1 (PA1) was found to be resistant to all tested antibiotics, except for polymixin B (MAR index: 0.92), whilst the *P. aeruginosa* 2 strain (PA2) was only resistant to piperacillin-tazobactam (MAR index: 0.08). 

Cinnamaldehyde was active against all strains of *P. aeruginosa*, including those with a multidrug resistance phenotype. MIC values were 0.25 mg/mL, 0.5 mg/mL and 1.0 mg/mL for PA1, PA2 and ATCC 27853, respectively. MBC values for PA1 and PA2 were similar to their respective MIC values, whilst the MBC observed for *P. aeruginosa* ATCC 27853 was 2.0-fold higher than its MIC value; these results indicate a bactericidal action for cinnamaldehyde. At the used concentration, the vehicle (2% DMSO in phosphate-buffered saline (PBS)) did not affect bacterial growth. 

We also assessed whether cinnamaldehyde causes adaptive phenotype in *P. aeruginosa*. The in vitro incubation of cinnamaldehyde did not induce such response in *P. aeruginosa* ATCC 27853 following 10 sequential passages. In contrast, this strain became tolerant to ciprofloxacin as MIC values for this antibiotic increased from 0.0625 to 1.0 µg/mL.

**Figure 1:**
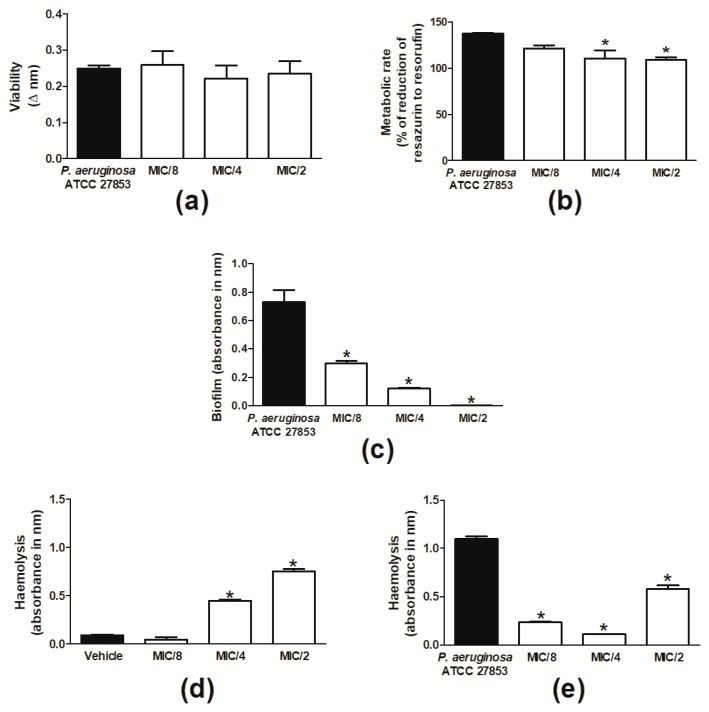
Effects of sub-inhibitory concentrations of cinnamaldehyde on *P. aeruginosa*. *P. aeruginosa* ATCC 27853 (**a**) viability (Δ nm) and (**b**) metabolic rate (as percentage (%) of reduction of resazurin to resorufin). (**c**) Biofilm formation and (**e**) haemolysis induced by *P. aeruginosa* ATCC 27853. Effect of sub-inhibitory concentrations of cinnamaldehyde on erythrocytes in the absence of bacteria (**d**). Cinnamaldehyde was tested at MIC/2, MIC/4 and MIC/8. Vehicle (2% DMSO in PBS)-treated bacteria were used as controls. * *p* < 0.05, differs from the vehicle-treated group. *n* = 3.

Then, the effects of sub-inhibitory concentrations of cinnamaldehyde were evaluated. Cinnamaldehyde did not alter the viability of *P. aeruginosa*, but decreased its metabolic rate when tested at MIC/4 and MIC/2 (Figure 1a,b). Cinnamaldehyde inhibited formation of biofilm by *P. aeruginosa* ATCC 27853 at all tested concentrations (MIC/8-MIC/2; Figure 1c). A similar effect was observed for this compound when assessed in *P. aeruginosa*-induced haemolysis (Figure 1e). On the other hand, in the absence of bacteria, cinnamaldehyde caused haemolysis per se, an effect that was observed when the compound was tested at MIC/4 and MIC/2 but not MIC/8 (Figure 1d).

### 2.2. Cinnamaldehyde Reduces the P. aeruginosa Population in Skin Wounds and Accelerates Their Healing

As sub-inhibitory concentrations of cinnamaldehyde were found to inhibit the metabolic rate of *P. aeruginosa*, and also its ability to form biofilm and cause haemolysis, the healing potential of the topical application of this compound at MIC/2 (0.5 mg/mL; 30 µL) was assessed in mouse skin wounds infected or not with *P. aeruginosa* ATCC 27853 (1.5 × 10^8^ cells/wound). 

**Figure 2:**
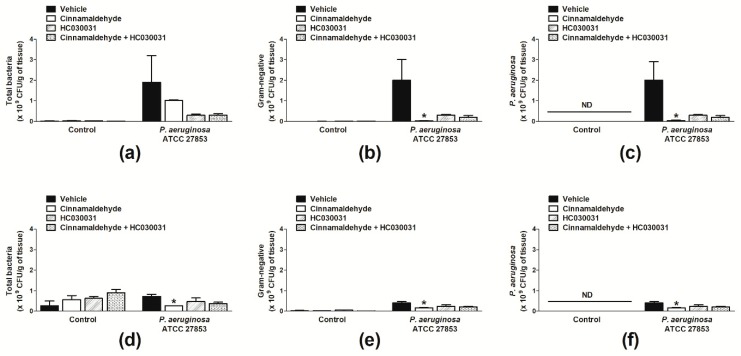
Effects of topical cinnamaldehyde or systemic TRPA1 antagonism on skin wound bacterial population. Total bacteria (**a**), Gram-negative bacteria (**b**) and *P. aeruginosa* (**c**) population in skin wound samples of infected and non-infected mice on day 4 post-skin excision. Total bacteria (**d**), Gram-negative bacteria (**e**) and *P. aeruginosa* (**f**) population in skin wound samples of infected and non-infected mice on day 7 post-skin excision. Animals received either sterile saline or *P. aeruginosa* ATCC 27853 following skin excision. Cinnamaldehyde (0.5 mg/mL; 30 µL, *n* = 8) or 2% DMSO in sterile saline (30 µL, *n* = 8) were topically applied once a day for seven days. The TRPA1 antagonist HC-030031 (30 mg/kg, i.p., *n* = 8) or vehicle (8% DMSO in saline, i.p., *n* = 8) were administered to animals receiving or not cinnamaldehyde. * *p* < 0.05, differs from vehicle-treated mice. ND: not detected.

We initially assessed the number of bacteria per wound following skin excision. Irrespective of treatment, animals not infected with *P. aeruginosa* presented higher numbers of total bacteria in their wounds on day 7 in comparison with day 4 post-surgery (Figure 2a,d). The skin wounds of these animals were primarily colonized by Gram-positive bacteria (Figure 2d,e). On the contrary, mice infected with *P. aeruginosa* and treated with vehicle just after the induction of the skin lesions mostly presented Gram-negative bacteria in their wounds; of note, these were all identified as *P. aeruginosa* (Figure 2a–f). 

The topical application of cinnamaldehyde significantly reduced the total Gram-negative and *P. aeruginosa* populations in the skin wounds of mice infected with this bacterium at both evaluated time points (Figure 2a–f); whilst increasing the number of Gram-positive bacteria in the absence of this pathogen at 7 d post-skin excision (Figure 2d). Similar responses were observed for mice treated with HC-030031 or cinnamaldehyde plus HC-030031 (Figure 2a–f).

**Figure 3:**
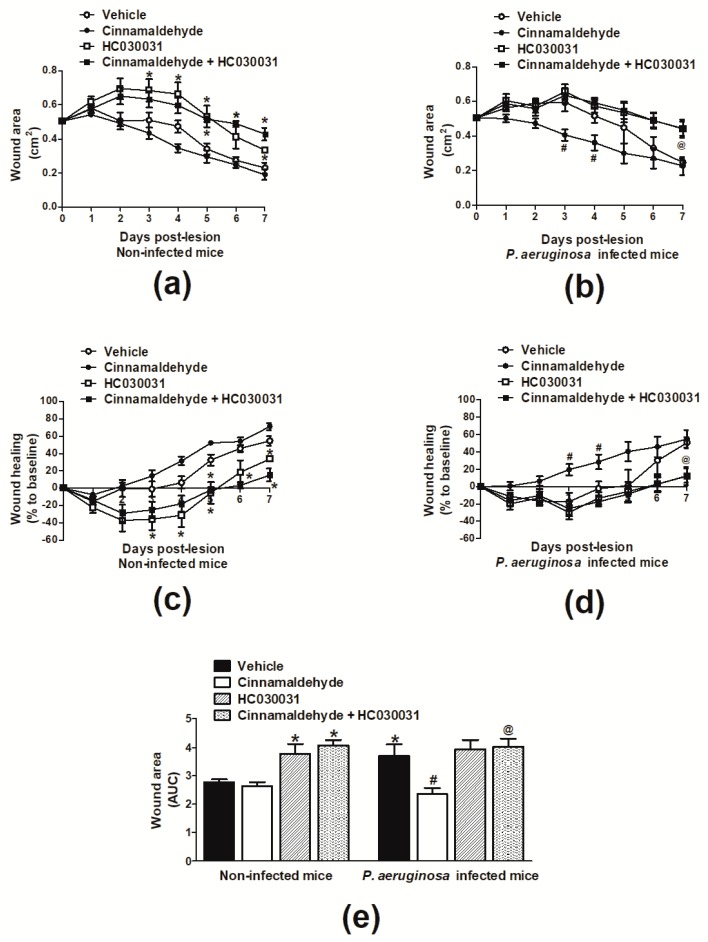
Effects of topical cinnamaldehyde or systemic TRPA1 antagonism on wound healing. Time course of (**a**) wound area and (**b**) healing in non-infected mice. Time course of (**c**) wound area and (**d**) healing in *P. aeruginosa*-infected mice. (**e**) Area under de curve (AUC) of wound area over a seven-day time-course. Animals received either sterile saline or *P. aeruginosa* ATCC 27853 following skin excision. Cinnamaldehyde (0.5 mg/mL; 30 µL, *n* = 6) or 2% DMSO in sterile saline (30 µL, *n* = 6) were topically applied once a day for sevn days. The TRPA1 antagonist HC-030031 (30 mg/kg, i.p., *n* = 6) or vehicle (8% DMSO in saline, i.p., *n* = 6) were administered to animals receiving or not cinnamaldehyde. * *p* < 0.05, differs from vehicle-treated non-infected mice; ^#^ *p* < 0.05, differs from vehicle-treated *P. aeruginosa* infected mice; ^@^ *p* < 0.05, differs from cinnamaldehyde-treated *P. aeruginosa* infected mice.

Figure 3a,c depicts the wound area and healing of animals not infected with *P. aeruginosa*. A slight enlargement of the wounded area was observed for all tested groups at 24 h post-skin lesion. In vehicle (2% DMSO in saline; 30 µL)-treated mice, healing was noted from day 4 post-wound induction. At this time point, lesion was reduced by 6.4% in comparison with the initial area (day 0), reaching 54.5% by day 7 post-lesion. In animals topically treated with cinnamaldehyde, healing was present from day 3 post-wound induction, as the compound diminished the area of lesion at this time point by 14.1% in comparison with the initial lesion. In the same group, wound was contracted by 31.3% and 62.3% at the 4th and 7th days post-lesion, respectively. 

Vehicle-treated *P. aeruginosa*-infected mice presented healing from day 6 post-induction of lesion, as the wounded area contracted by 34.5% at this time point and by 51.2% at the end of the observation period (Figure 3b,d). Infected animals receiving cinnamaldehyde exhibited healing from the 2nd day following wound induction; with wounds presenting 14.9% contraction at this time point, and reduction of the wounded area by 54.8% on day 7 when compared with the initial lesion (Figure 3b,d). 

Differences in wound contraction between groups can be better evidenced in Figure 3e, in which the area under the curve (AUC) over seven days is plotted. Vehicle-treated mice infected with *P. aeruginosa* presented larger wounded areas in comparison with non-infected controls. Cinnamaldehyde significantly reduced (36%) the skin lesions of animals infected with *P. aeruginosa* without affecting the wounded area of non-infected mice.

**Figure 4:**
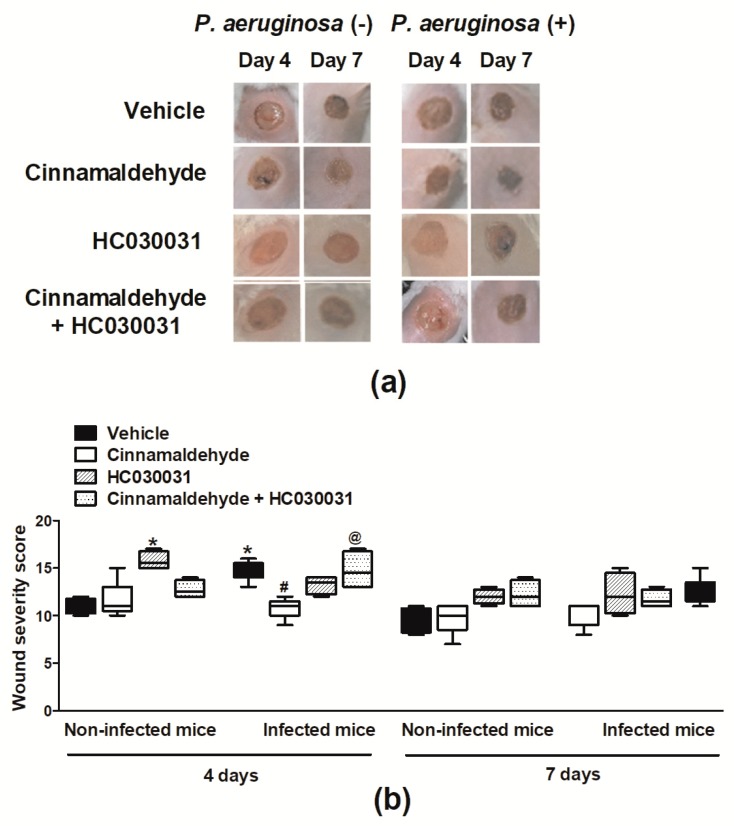
Effects of topical cinnamaldehyde or systemic TRPA1 antagonism on wound severity. Wound severity was macroscopically evaluated. Representative photographs (**a**) and severity scores (**b**) of the wounds on days 4 and 7 post-skin excision. Animals received either sterile saline or *P. aeruginosa* ATCC 27853 following skin excision. Cinnamaldehyde (0.5 mg/mL; 30 µL, *n* = 6) or 2% DMSO in sterile saline (30 µL, *n* = 6) were topically applied once a day for over seven days. The TRPA1 antagonist HC-030031 (30 mg/kg, i.p., *n* = 6) or vehicle (8% DMSO in saline, i.p., *n* = 6) were administered to animals receiving cinnamaldehyde or not. * *p* < 0.05, differs from vehicle-treated non-infected mice; ^#^ *p* < 0.05, differs from vehicle-treated *P. aeruginosa* infected mice; ^@^ *p* < 0.05, differs from cinnamaldehyde-treated *P. aeruginosa* infected mice.

The severity of the wounds was evaluated macroscopically (based on wound healing, exudate, oedema, surrounding tissue color, debridement tissue type and necrosis) on days 4 and 7 post-skin excision. *P. aeruginosa* infection increased the severity of vehicle-treated wounds in comparison with non-infected controls (Figure 4a,b; day 4). The wound severity of *P. aeruginosa* infected mice was significantly reduced by cinnamaldehyde (Figure 4a,b). On the other hand, this compound did not affect the severity of wounds of non-infected animals (Figure 4a,b).

### 2.3. Cinnamaldehyde-Induced Wound Healing Is Prevented by Transient Receptor Potential Ankyrin 1 (TRPA1) Antagonism in Mice Infected with P. aeruginosa

The contribution of TRPA1, a well-known target for cinnamaldehyde, in skin wound healing was evaluated by administering the TRPA1 antagonist HC-030031 (30 mg/kg; intraperitoneal (i.p.), once a day) from day 1 post-wound induction, at 1 h prior to topical application of cinnamaldehyde or vehicle. Figure 3a,c demonstrates that animals not infected with *P. aeruginosa* but treated with both cinnamaldehyde and HC-030031, exhibited healing and reduction of the wounded area from the 6th day post-lesion, reaching a 15.1% contraction at the end of the observation period (7th day). A similar contraction course was observed in those receiving HC-030031 only, as their wounds were reduced from day 6 post-lesion (18.3%), reaching 33.6% contraction at the 7th day. AUC analysis in mice not infected with *P. aeruginosa*, indicated that HC-030031 treatment alone, or in combination with cinnamaldehyde, increases the wound area over time (Figure 3e). 

On the other hand, in mice infected with *P. aeruginosa*, cinnamaldehyde-induced healing was prevented by treatment with HC-030031 (Figure 3b,d,e). Animals of this group did not exhibit healing and only presented a 2.9% contraction of the wounded area by the end of the seven-day course. A similar profile was observed in infected animals treated solely with the TRPA1 antagonist. 

Additionally, the macroscopic evaluation of the skin lesions (Figure 4a,b) indicated that HC-030031 *per se*, increases the severity of the wounds of mice that were not infected with *P. aeruginosa*. On the contrary, this compound did not affect cinnamaldehyde actions in non-infected mice (Figure 4a,b). Analysis of the wounds of animals infected with *P. aeruginosa* demonstrated that HC-030031 treatment blocks cinnamaldehyde-induced protection (Figure 4a,b). However, the skin wounds of infected mice topically applied with vehicle were not affected by the systemic TRPA1 antagonism (Figure 4a,b).

Effects of the systemic treatment with HC-030031 on wound bacterial colonization were also evaluated. Figure 2a–f demonstrates that TRPA1 antagonism reduces the numbers of Gram-negative and Gram-positive bacteria in the wound beds of *P. aeruginosa*-infected mice; whilst increasing the numbers of bacteria in those not infected with this pathogen. These effects were not significant. Also, HC-030031 treatment did not alter the topical effects of cinnamaldehyde on wound bacterial colonization (Figure 2a–f). 

### 2.4. Cinnamaldehyde Reduces the Production of Key Inflammatory Mediators in the Wound Beds of P. aeruginosa-Infected Mice

**Figure 5:**
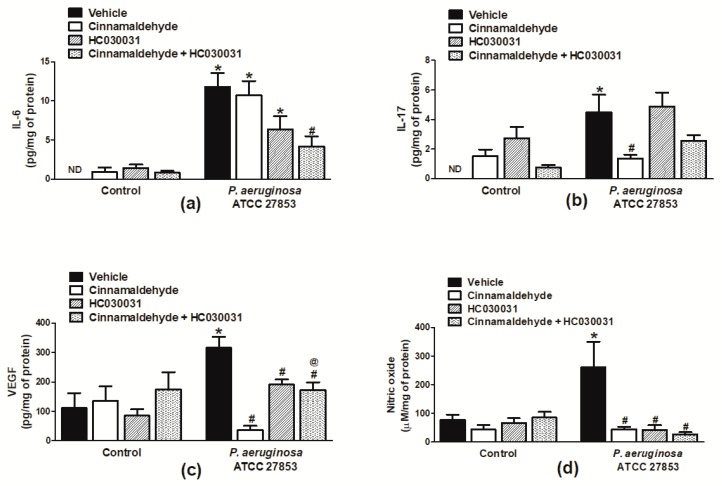
Inflammatory mediator release in cinnamaldehyde-treated mouse skin wounds. The levels of (**a**) IL-6, (**b**) IL-17, (**c**) VEGF and (**d**) NO in skin wounds of animals infected or not with *P. aeruginosa* ATCC 27853. Cinnamaldehyde (0.5 mg/mL; 30 µL, *n* = 6) or 2% DMSO in sterile saline (30 µL, *n* = 6) were topically applied once a day for four days. The TRPA1 antagonist HC-030031 (30 mg/kg, i.p., *n* = 6) or vehicle (8% DMSO in saline, i.p., *n* = 6) were administered to animals receiving or not cinnamaldehyde. * *p* < 0.05, differs from vehicle-treated non-infected mice; ^#^ *p* < 0.05, differs from vehicle-treated *P. aeruginosa* infected mice; ^@^ *p* < 0.05, differs from cinnamaldehyde-treated *P. aeruginosa*-infected mice. ND: not detected.

The production of range of inflammatory mediators underlie different stages of the healing process; therefore, the effects of the topical application of cinnamaldehyde on the production of interleukin-6 (IL-6) and 17 (IL-17), vascular endothelial growth factor (VEGF) and nitric oxide (NO), all known to play a role in wound healing [19], were analysed. The data depicted in Figure 5 demonstrate that, by day 4 post-wound induction, *P. aeruginosa* significantly increased the production of all the assessed inflammatory mediators in vehicle-treated mice in comparison with their non-infected counterparts (Figure 5a–d). Cinnamaldehyde impaired the production of IL-17, VEGF and NO, with percentages of inhibition of 69.4, 88.4% and 83.3%, respectively (Figure 5b–d). Cinnamaldehyde did not alter IL-6 production in the same mice (Figure 5a). Similarly, this compound had no significant effects on the inflammatory mediator production of mice not infected with *P. aeruginosa* (Figure 5a–d).

### 2.5. Cinnamaldehyde’s Inhibitory Effects on the Production of Inflammatory Mediators in P. aeruginosa-Infected Skin Wounds Partially Depends on TRPA1 Activation

The contribution of TRPA1 activation to cinnamaldehyde inhibitory actions in the inflammatory mediator release in wound beds infected with *P. aeruginosa* was assessed in mice systemically treated with HC-030031. It was observed a marked reversion of cinnamaldehyde effects in regards of VEGF and IL-17, but not IL-6 and NO production in these mice (Figure 5a–d). Also, this compound did not alter the inflammatory mediator levels in mice topically receiving cinnamaldehyde, in the absence of *P. aeruginosa*. HC-030031 *per se*, significantly inhibited VEGF production (39.8%; Figure 5c) in *P. aeruginosa*-infected wounds whilst increasing IL-17 levels in those not infected with this pathogen (Figure 5b). No effects were observed on NO, IL-6 or VEGF production in mice not infected with *P. aeruginosa* and administered HC-030031 only (Figure 5a,c,d).

## 3. Discussion

Skin wound healing can be impaired or delayed by the colonization of the wounds by microorganisms such as *P. aeruginosa*, commonly resistant to the available antibiotic therapy [8,9]. In this context, an ideal therapy for infected wounds should not only inhibit the pathogen, but also present healing activity. 

Herein, cinnamaldehyde was antimicrobial against *P. aeruginosa* strains, including those with a multidrug resistance phenotype; and it also attenuated bacterial virulence. These findings are supported by recent evidence on that this compound at MIC/2, disrupts pre-formed biofilms of *P. aeruginosa* through inhibition of intracellular signalling processes involved in the control of biofilm formation by this pathogen [15]. Similarly, a cinnamaldehyde-enriched oil exhibited anti-biofilm activity equivalent to that found in our study [20]. Of note, anti-biofilm strategies have been considered interesting novel therapeutic approaches to prevent or disrupt biofilms in persistent infections by *P. aeruginosa* [21,22,23]. 

Biofilm formation is an important mechanism of bacterial colonization of skin wounds [24]. Therefore, we next explored the effects of the topical application of cinnamaldehyde on *P. aeruginosa*-infected skin wounds in mice. Daily topical application of cinnamaldehyde reduced the load of *P. aeruginosa* in skin wounds and also promoted faster healing of these wounds. Overall, cinnamaldehyde did not affect the area of the wounds not infected by *P. aeruginosa*, although a healing response was observed one day earlier in these mice in comparison with vehicle controls. In addition, this compound did not affect the number of other bacteria colonizing these lesions. These results suggest that cinnamaldehyde healing effects may dependent on the pathogen present in the wounds, as different mechanisms may be involved in the host responses to such infections. Of note, the systemic administration of cinnamaldehyde accelerated skin wound healing rats [18], suggesting that the route of administration of this compound may also interfere with healing. 

TRPA1 is a well-documented target of cinnamaldehyde as an agonist [25]. It is a member of the transient receptor potential (TRP) family expressed on neuronal and non-neuronal cells and it has been pointed as a key mediator of skin perfusion and also as a sensor of bacterial infection [9,10,26,27,28]. Interestingly, TRPA1 activation in vitro induced the mRNA expression of genes involved in the control of keratinocyte proliferation and differentiation [29]. Our data demonstrated that the repeated systemic administration of HC-030031 prevents cinnamaldehyde-induced healing in mice infected with *P. aeruginosa*. Of note, the studies on TRPA1 as a bacterial sensor are few and have mainly concentrated on *E. coli* signalling [26,27]; thus, we present herein the first evidence that TRPA1 mediates host–*P. aeruginosa* interactions in vivo. 

Interestingly, mice administered with HC-030031 that were not infected with this pathogen presented larger lesions in comparison with the control group; this suggests that the endogenous activation of TRPA1 is important to healing, even in the absence of *P. aeruginosa*. Few reports have assessed the role of TRPA1 in wound healing. Yang and collaborators [30] indicated that burn patients with broader skin lesion areas express increased levels of TRPA1. It was also shown that the loss of TRPA1 signalling reduces inflammation and improves corneal healing in mice with chemical burns [31]. Another study by Hayashi et al. [32] suggested that TRPA1 activation inhibits the repair of the stomach epithelial wounds. All this evidence and the data gathered herein allow us to conclude that TRPA1’s role in wound healing may depend on the tissue type and stimuli.

An analysis of our model demonstrates that, at by 4 post-infection, *P. aeruginosa*-infected wounds are characterized by an inflammatory milieu in comparison with non-infected controls. This response included the upregulation of IL-6 and 17, IL-17, VEGF and NO, all known to play a role in wound healing [19]. It may seem counterintuitive that VEGF is upregulated in chronic wounds, but this has been observed in other chronic wounds, such as aphthous ulcers [33]. The effect of VEGF in healing may be context-specific [34]. In chronic inflammation, VEGF may have a preferential effect on vascular leak over revascularization. Therefore, angiogenesis inhibition might assist re-epithelialisation [33]. 

Herein, it was found that cinnamaldehyde reduces the production of key inflammatory mediators (IL-17, VEGF and NO) in the wound beds of *P. aeruginosa*-infected mice. Cinnamaldehyde anti-inflammatory effects are not novel, and different studies have demonstrated its ability to reduce NO and pro-inflammatory cytokine generation upon LPS stimuli [26,35,36,37]. However, its modulatory role on VEGF expression is unclear. Contrary to the data presented herein, a recent study suggested that the systemic treatment with cinnamaldehyde induces skin wound healing in diabetic mice by increasing VEGF levels [18]. It is possible that cinnamaldehyde effects on VEGF release during infection depend on the stimuli (infection versus diabetes) and treatment schemes (intraperitoneal versus topical administration). Of note, cinnamaldehyde presented no significant effects on the bacterial numbers or the levels of inflammatory mediators in skin lesions t infected with *P. aeruginosa*.

The contribution of TRPA1 activation to cinnamaldehyde inhibitory actions in inflammatory mediator release in wound beds infected with *P. aeruginosa* was also assessed in mice systemically treated with HC-030031. VEGF and IL-17 levels in the skin wounds of mice infected with *P. aeruginosa* and treated with cinnamaldehyde, were partially attenuated by the systemic administration of the TRPA1 antagonist HC-030031; a drug that did not affect the number of bacteria in cinnamaldehyde-applied lesions. Interestingly, TRPA1 antagonism effects on NO release were similar to that of cinnamaldehyde. *P. aeruginosa*-derived LPS was recently shown to activate TRPA1 in vitro, although in a smaller extent than *E. coli* LPS [27]. These evidences suggest that cinnamaldehyde and HC-030031 effects on NO release may be due to the ability of cinnamaldehyde and HC-030031 to compete with *P. aeruginosa* LPS for a binding site on TRPA1. The partial recovery of IL-17 and VEGF production following TRPA1 antagonism in animals topically applied with cinnamaldehyde, indicates a complex scenario in terms of activation sites, which remains to be further elucidated. Also, considering the TRPA1 expression on different cells involved in skin wound healing such as neurones, keratinocytes and immune cells [38], it is not yet known in which cells these molecules (LPS, cinnamaldehyde and HC-030031) are preferentially binding to in the wound beds infected by *P. aeruginosa* in order to delay or promote skin healing. Further in vitro and in vivo studies are necessary to determine the specific actions of cinnamaldehyde as well as the role of TRPA1 on each cell type involved in skin healing (fibroblast, keratinocyte and endothelial cell culture) under infected and non-infected conditions. In this context, further histological and immunohistochemical analysis would be also valuable.

**Figure 6:**
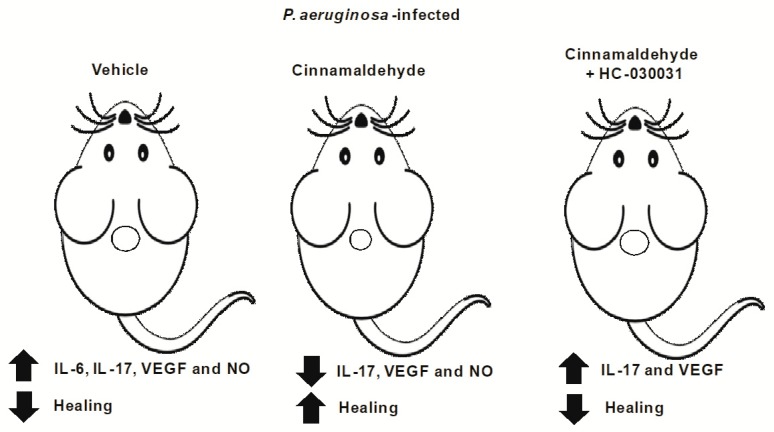
Summary of cinnamaldehyde effects in the wound healing of mice infected with *P. aeruginosa*. Mice infected with *P. aeruginosa* have delayed healing, associated with increased production of inflammatory mediators in the wound beds in response to this bacterial infection. The topical application of cinnamaldehyde reduces the bacterial load in the wound tissue and promotes wound healing, as denoted by reduction of the wounded area. This effect is associated with diminished levels of IL-17, VEGF and NO in the wound beds. The systemic TRPA1 antagonism by HC-030031 prevents cinnamaldehyde’s pro-healing action by increasing IL-17 and NO levels.

Overall, our data demonstrate that the repeated topical application of cinnamaldehyde promotes faster healing of skin wounds infected by *P. aeruginosa* by decreasing bacterial colonization and attenuating the production of key inflammatory mediators of tissue regeneration such as IL-17, VEGF and NO (Figure 6). The results also indicate that this anti-inflammatory effect is partially mediated by TRPA1 activation, although the cells involved in this process remain to be determined. Modification of both bacterial and host factors will likely be required for successful wound healing, and agents that disrupt *P. aeruginosa* virulence without causing resistance might be especially valuable [39,40]. We suggest that topical formulations (gel, nanoemulsion or aerosol formulations) containing sub-inhibitory concentrations of cinnamaldehyde may be a useful tool to treating skin infections induced by *P. aeruginosa*. 

## 4. Materials and Methods

### 4.1. Bacterial Cultures

Two clinical isolates of *P. aeruginosa* (PA1 and PA2) and the standard *P. aeruginosa* strain ATCC 27853 (all obtained from the culture collection sector of the Universidade CEUMA) were used in the study. Their susceptibility to antimicrobials was assessed in the automated VITEK^®^ 2 system (BioMérieux Clinical Diagnostics, Lombard, IL, USA) and data interpretation was performed as recommended by the Clinical Laboratory Standards Institute [41]. The multiple antibiotic resistance (MAR) index was calculated as previously described [42], by using the formula MAR = x/y, where “x” corresponds to the number of antibiotics to which the isolate demonstrated resistance; and “y” to the total number of antibiotics tested. 

### 4.2. In Vitro Studies

#### 4.2.1. Determination of MIC and MBC and Analysis of Bacterial Tolerance to Drug

The antimicrobial activity of *trans*-cinnamaldehyde (Sigma-Aldrich^®^, St. Louis, MO, USA; 99% purity) was determined by the microdilution method [41]. For this, each bacterial strain was grown on Müeller‒Hinton Agar (MHA) plates at 37 °C for 24 h, and suspended in saline solution (~1.5 × 10^8^ CFU/mL). The minimum inhibitory concentrations (MICs) were determined by the incubation for 24 h at 37 °C, of 10 μL of each bacterial suspension with Müeller‒Hinton (MH) broth containing different concentrations of cinnamaldehyde (62.5–2000 μg/mL) or ciprofloxacin (0.06–256 µg/mL; positive control). Sterile dimethyl sulfoxide (DMSO; 2% in phosphate-buffered saline; PBS) was used as negative control. Moreover, the effects of cinnamaldehyde on bacterial viability and metabolism were assessed and calculated by addition of PrestoBlue^®^ reagent (1:10; Life Technologies, São Paulo, SP, Brazil), according with the manufacturer´s instructions. 

For determining the minimum bactericidal concentrations (MBCs), at the end of the MIC experiments, 10 µL of the cultures were streaked onto MHA and incubated for 24 h at 37 °C. 

The ability of cinnamaldehyde to induce tolerance in *P. aeruginosa* was also assessed. For this, the reference strain ATCC 27853 was used. Briefly, 1 mL of the bacterial suspensions was incubated with MH broth containing sub-inhibitory concentrations of cinnamaldehyde or ciprofloxacin (MIC/2), for 24 h at 37 °C, as previously described [42]. Vehicle-treated bacteria (2% DMSO in PBS) were used as negative controls.

#### 4.2.2. Biofilm Formation

The anti-biofilm formation effects of cinnamaldehyde were assessed in 96-well cell culture plates, as described by Ferro et al. [42]. For this, 10 µL/well of bacterial suspensions (prepared as described above) were incubated with sub-inhibitory concentrations of the compound (MIC/2 and MIC/4) and 200 μL of Luria-Bertani (LB) broth, at 37 °C, for 24 h. Then, the wells were washed with PBS (3×). The formed biofilm was stained with 5% crystal violet for 10 min at room temperature, and immediately solubilised with methanol (200 µL/well; 100%). The absorbance was read at 570 nm and taken as an index of biofilm formation. Vehicle (2% DMSO in PBS)-treated bacteria and broth without bacteria were used as positive and negative controls, respectively.

#### 4.2.3. Haemolysis

Cinnamaldehyde effects on haemolysis were analysed as previously described [42]. The study was reviewed and approved by the Human Research Ethics Committee of the Universidade CEUMA (CEP-UNICEUMA; protocol number 1.336.315) and was performed in accordance with the Declaration of Helsinki 1975, as revised in 2008.

The samples (2.5 mL of blood) were collected in heparinised tubes and the erythrocytes were obtained by centrifugation (1500 rpm, 10 min). Two hundred microliters of BHI broth containing erythrocytes (2%) were incubated with 10 µL of each bacterial suspension (~1.5 × 10^8^ CFU/mL) and sub-inhibitory concentrations of cinnamaldehyde (MIC/2 and MIC/4) or vehicle (2% DMSO in PBS). After 24 h of incubation at 37 °C, the samples were centrifuged and the supernatant (100 µL/per sample/well) was transferred to a 96-well plate. Absorbance was read at 550 nm and taken as index of haemolytic activity. 

### 4.3. In Vivo Experiments

#### 4.3.1. Animals

Non-fasted outbred female Swiss mice (four months old; ~35 g), kept in a climatically controlled environment (room temperature of 22 ± 2 °C, humidity of around 60% and 12:12 h light‒dark cycle), were obtained from the animal facility of Universidade CEUMA (UNICEUMA). All procedures were approved by the Ethics Committee of UNICEUMA (protocol number 100/13) and carried out in accordance with the Brazilian Society for Animal Welfare.

#### 4.3.2. Induction of Skin Wounds

Skin wounds were induced in mice anaesthetised with a mixture of ketamine (50 mg/kg; Dopalen, Ceva, Paulínia, SP, Brazil) and xylazine (2 mg/kg; Dopaser, Hertape Calier, Belo Horizonte, MG, Brazil) by intraperitoneal (i.p.) route. Following anaesthesia, the dorsal skin was shaved and an asepsis was then, performed with 70% ethanol. A single full-thickness dorsal skin excision of 0.8 cm of diameter was made in each mouse. Then, the resulting wound was inoculated with *P. aeruginosa* (~1.5 × 10^8^ UFC/mL; 30 µL; *n* = 6–8). Control mice received a similar volume of sterile PBS (vehicle; *n* = 6–8). In order to minimize external contamination, each wound was dressed and the dressings were changed once daily just before the topical treatments. The wounds were observed for seven days. A macroscopic evaluation of the wounds was performed on days 4 and 7 post-skin excision by analysing different parameters to which a score was attributed as shown in Table 2. The summation of the individual scores for each mouse was taken as index of wound severity; i.e., the higher the score, the more severe is the wound. The wound area (cm^2^) was daily measured and recorded. Reductions of the wounded area were taken as index of wound healing. The results are expressed as the percentage (%) of healing in relation to baseline wound areas.

#### 4.3.3. Pharmacological Treatments

Twenty-four hours following *P. aeruginosa* infection, mice had either sterile cinnamaldehyde (0.5 mg/mL; 30 µL; *n* = 6–8) or the vehicle (2% DMSO in saline; *n* = 6–8) applied topically. In order to investigate the involvement of TRPA1 in the topical effects of cinnamaldehyde, mice received daily treatment with the TRPA1 antagonist HC-030031 (30 mg/kg; i.p.; *n* = 6–8), 24 h prior to cinnamaldehyde application. Vehicle-injected mice were used as controls (8% DMSO in saline; i.p.; *n* = 6–8). 

#### 4.3.4. Analysis of Wound Bacterial Colonization

In order to determine the bacterial population in mouse wound beds (*n* = 8, per time point), animals were culled by an i.p. overdose of ketamine (100 mg/kg) and xylazine (2 mg/kg), followed by cervical dislocation, on days 4 and 7 post-*P. aeruginosa* infection. The wounds were excised and half of each sample was then weighed and placed in sterile tubes containing 1 mL of saline. Samples were vortexed (five times, for 30 s each), and the resulting suspensions were serially diluted (1:10) and plated on to sheep blood (for determination of total aerobic and facultative bacteria) and MacConkey (for determination of aerobic and facultative Gram-negative bacteria) agars. The plates were incubated at 37 °C for 24 h. To determine the population of *P. aeruginosa*, the isolates grown on MacConkey agar, which presented colonies with green colour and grape-like odour and were lactose-negative, were tested for oxidase activity with 1% tetramethyl *p*-phenylenediamine dihydrochloride (BD BBL DrySlide Oxidase). The numbers of total, Gram-negative, and *P. aeruginosa* bacteria were determined and the results expressed as CFU/g of tissue. 

#### 4.3.5. Sample Preparation for Analysis of Inflammatory Mediators in Wounds

Samples (half of the excised wound) obtained on day 4 post-infection were homogenized in 1 mL of ice-cold PBS containing protease inhibitors (cOmplete™, EDTA-free Protease Inhibitor Cocktail; Sigma-Aldrich; São Paulo, SP, Brazil), by using a tissue lyser (6 cycles of 30 s each, 4000 r.p.m.; between cycles, samples were kept on ice for 20 s; TissueLyser LT; Qiagen; São Paulo, SP, Brazil). The homogenates were then centrifuged at 1000 r.p.m., for 10 min, at 4 °C. The supernatant was collected and used for the measurements of NO, VEGF and cytokine levels (IL-6, IL-17) in the wound samples. The protein content of each supernatant was determined by using Bradford reagent, according with the manufacturer´s instructions (Sigma-Aldrich; São Paulo, SP, Brazil).

#### 4.3.6. Wound Levels of Nitric Oxide

The NO_3_^−^ content was reduced to NO_2_^−^ by incubating 80 µL of sample supernatant with 20 µL of 1U/mL of nitrate reductase (Sigma-Aldrich; São Paulo, SP, Brazil) and 10 µL of 1 mM of NADPH (Sigma-Aldrich; São Paulo, SP, Brazil) for 30 min at 37 °C [26]. Then, 100 µL of Griess reagent (5% *v*/*v* H_3_PO_4_ containing 1% *w*/*v* sulfanilic acid and 0.1% *w*/*v* *N*-1-napthylethylenediamine; Sigma-Aldrich; São Paulo, SP, Brazil) and further incubated for another 30 min at 37 °C. Absorbance was read at 550 nm using a spectrophotometer (Plate reader MB-580; Heales, Shenzhen, China). The absorbance of each sample was subtracted of background reading and compared with those of a standard curve (0–300 µM sodium nitrite). The results are expressed as NO_x_ levels per mg of tissue protein (µM/mg of protein).

#### 4.3.7. VEGF and Cytokine Measurements

Wound levels of VEGF were measured by using a commercial Mouse VEGF ELISA Kit for tissue lysates (Sigma-Aldrich; São Paulo, SP, Brazil). Tissue cytokine (IL-6 and IL-17) levels were evaluated by using mouse cytometric bead array (CBA) cytokine kits (BD Biosciences, São Paulo, SP, Brazil) and analysed on a Facscalibur cytometer flow cytometer (BD Biosciences‒Immunocytometry Systems, San Diego, CA, USA) as described by Mendes et al. [26]. All assays were performed according to the manufacturer’s instructions. Results are expressed as picograms of sample per milligram (pg/mg) of tissue. 

### 4.4. Statistical Analysis

The results are presented as the mean ± standard error (SE). The in vitro assays were performed in duplicate in four independent experiments. An *n* of 6–8 animals per group was used in vivo, from three independent experiments. Statistical comparison between groups was performed in the software GraphPad Prism version 5.0 (GraphPad Software Inc., San Diego, CA, USA) by using one-way or two-way analysis of variance followed by the Bonferroni test. The results of the severity score analysis are expressed as the median (minimum-maximum) values and were analysed using Kruskal-Wallis test followed by Dunn´s test for multiple comparisons. *p* < 0.05 was considered significant.

## Figures and Tables

**Table 1:** Antibiotic susceptibility profile of *P. aeruginosa*.

Strain	Antibiotic											MAR
	AMI	AZT	CEP	CET	CIP	GEN	LEV	IMI	MER	PIP/TAZ	POL	
*P. aeruginosa* ATCC 27853	S	S	S	S	S	S	S	S	S	S	S	0
*P. aeruginosa* 1	R	R	R	R	R	R	R	R	R	R	S	0.92
*P. aeruginosa* 2	S	S	S	S	S	S	S	S	S	R	S	0.08

AMI: amycacin; AZT: aztreonam; CEP: cefepime; CET: ceftazidime; CIP: ciprofloxacin; GEN: gentamicin; LEV: levofloxacin; IMI: imipenem; MER: meropenem; PIP/TAZ: piperacillin-tazobactam; POL: polymyxin B; R: resistant; S: susceptible; Multiple antibiotic resistance (MAR) index.

**Table 2:** Macroscopic evaluation of the wounds.

Evaluated Parameters	Range	Score
Wound healing (% in relation to baseline wound area)	0–20%	0
	21–40%	1
	41–60%	2
	61–80%	3
	81–100%	4
	101–120%	5
	121–140%	6
	141–160%	7
	>160%	8
Exudate	No exudate	0
	Light	2
	Moderate	3
	Heavy	4
Exudate type	No exudate	0
	Blood	1
	Serosanguineous	2
	Serous	3
	Purulent	4
Oedema	No oedema	0
	Mild	1
	Moderate	2
	Severe	3
Surrounding skin tissue colour	Normal	0
	Red	1
	White or hypopigmented	2
	Dark red or purple	3
	Black or hyperpigmented	4
Debridement tissue type	Epithelial tissue	0
	Granulation tissue	1
	Granulation tissue	2
	Necrotic tissue	3
Amount of necrotic tissue (% in relation to the total wound area)	Absence of necrosis	0
	<25%	1
	25–49%	2
	50–75%	3
	76–100%	4

The summation of each score for each mouse was taken as index of wound severity. The higher the score, the more severe is the wound.
